# mRNA and miRNA Expression Analyses of the *MYC*/*E2F*/miR-17-92 Network in the Most Common Pediatric Brain Tumors

**DOI:** 10.3390/ijms22020543

**Published:** 2021-01-07

**Authors:** Renata Gruszka, Krzysztof Zakrzewski, Paweł Piotr Liberski, Magdalena Zakrzewska

**Affiliations:** 1 Department of Molecular Pathology and Neuropathology, Medical University of Lodz, Pomorska 251, 92-216 Lodz, Poland; pawel.liberski@umed.lodz.pl (P.P.L.); magdalena.zakrzewska@umed.lodz.pl (M.Z.); 2 Biobank Lab, Department of Molecular Biophysics, Faculty of Biology and Environmental Protection, University of Lodz, Pomorska 139, 90-235 Lodz, Poland; 3 Department of Neurosurgery, Polish Mother Memorial Hospital Research Institute in Lodz, Rzgowska 281/289, 93-338 Lodz, Poland; krzysztof.zakrzewski@iczmp.edu.pl

**Keywords:** OncomiR-1, brain tumor, miR-106a-363, miR-106b-25, miR-17-92, microRNA, medulloblastoma, ependymoma, pilocytic astrocytoma

## Abstract

Numerous molecular factors disrupt the correctness of the cell cycle process leading to the development of cancer due to increased cell proliferation. Among known causative factors of such process is abnormal gene expression. Nowadays in the light of current knowledge such alterations are frequently considered in the context of mRNA–miRNA correlation. One of the molecular factors with potential value in tumorigenesis is the feedback loop between *MYC* and *E2F* genes in which miR-17-5p and miR-20a from the miR-17-92 cluster are involved. The current literature shows that overexpression of the members of the OncomiR-1 are involved in the development of many solid tumors. In the present work, we investigated the expression of components of the *MYC*/*E2F*/miR-17-92 network and their closely related elements including members of *MYC* and *E2F* families and miRNAs from two paralogs of miR-17-92: miR-106b-25 and miR-106a-363, in the most common brain tumors of childhood, pilocytic astrocytoma (PA), WHO grade 1; ependymoma (EP), WHO grade 2; and medulloblastoma (MB), WHO grade 4. We showed that the highest gene expression was observed in the *MYC* family for *MYCN* and in the *E2F* family for *E2F2*. Positive correlation was observed between the gene expression and tumor grade and type, with the highest expression being noted for medulloblastomas, followed by ependymomas, and the lowest for pilocytic astrocytomas. Most members of miR-17-92, miR-106a-363 and miR-106b-25 clusters were upregulated and the highest expression was noted for miR-18a and miR-18b. The rest of the miRNAs, including miR-19a, miR-92a, miR-106a, miR-93, or miR-25 also showed high values. miR-17-5p, miR-20a obtained a high level of expression in medulloblastomas and ependymomas, while close to the control in the pilocytic astrocytoma samples. miRNA expression also depended on tumor grade and histology.

## 1. Introduction

miRNA molecules are involved in the post-transcriptional regulation of gene expression, and changes in their activity are associated with development of cancer by modulating oncogenic and/or tumor suppressor pathways. Moreover, miRNAs are still being studied as useful biomarkers, promising a valuable diagnostic tool useful in defining the prognosis and helpful in identifying the targeted therapy strategies [1,2]. One of the most recognized miRNA families is the miR-17-92 cluster (OncomiR-1), whose particular members demonstrate oncogenic functions influencing cell proliferation, apoptosis, and neoplastic angiogenesis [3]. OncomiR-1 contains six miRNAs: miR-17, miR-18, miR-19a, miR-20, miR-19b, and miR-92 derived from a common pri-mRNA localized in the *MIR17HG*/*C13orf25* human gene located on chromosome 13. The cluster has two paralogs: the miR-106b-25 and the miR-106a-363, which comprise miR-106b, miR-93, and miR-25 in the *MCM7* gene on chromosome 7 and miR-106a, miR-18b, miR-19b-2, miR-20b, miR-92a-2, and miR-363 on the X chromosome, respectively [3,4]. The most common element of these three clusters is their origin. miR-17-92 and its paralogs were probably created through tandem genetic duplication of individual cluster members, followed by duplication of entire clusters and subsequent loss of individual miRNAs [4]. This hypothesis was confirmed by the possibility of grouping specific miRNAs on the basis of sequence homology into four miRNA families: miR-17 family, (miR-17-5p, miR-20a, miR-20b, miR-106a, miR-106b, miR-93), miR-18 family (miR-18a, miR-18b), miR-19 family (miR-19a, miR-19b-1, miR-19b-2), and miR-92 family (miR-92a-1, miR-92a-2, miR-25, miR-363) [4].

Experimental studies have shown that there is a negative feedback loop among members of the miR-17-92 cluster and *E2F* and *MYC* transcription factors [5,6]. Many mathematical models of the *MYC*/*E2F*/miR-17-92 network were created, estimating how overexpression of the miR-17-92 cluster affects different types of cancers [5,7,8]. The consecutive studies provide evidences that the overexpression of miR-17-92 members is involved in the development of many solid tumors, including lung [9], breast [10], colon [11], hepatocellular [12], and stomach cancer [13]. Their essential role in adipocyte differentiation [14], lung development [15], angiogenesis [16], tumorigenesis [17], and heart development [18] was also underlined.

## 2. Results

### 2.1. MYC and E2F Gene Expression Is Connected with Tumor Type and Grade

**Figure 1:**
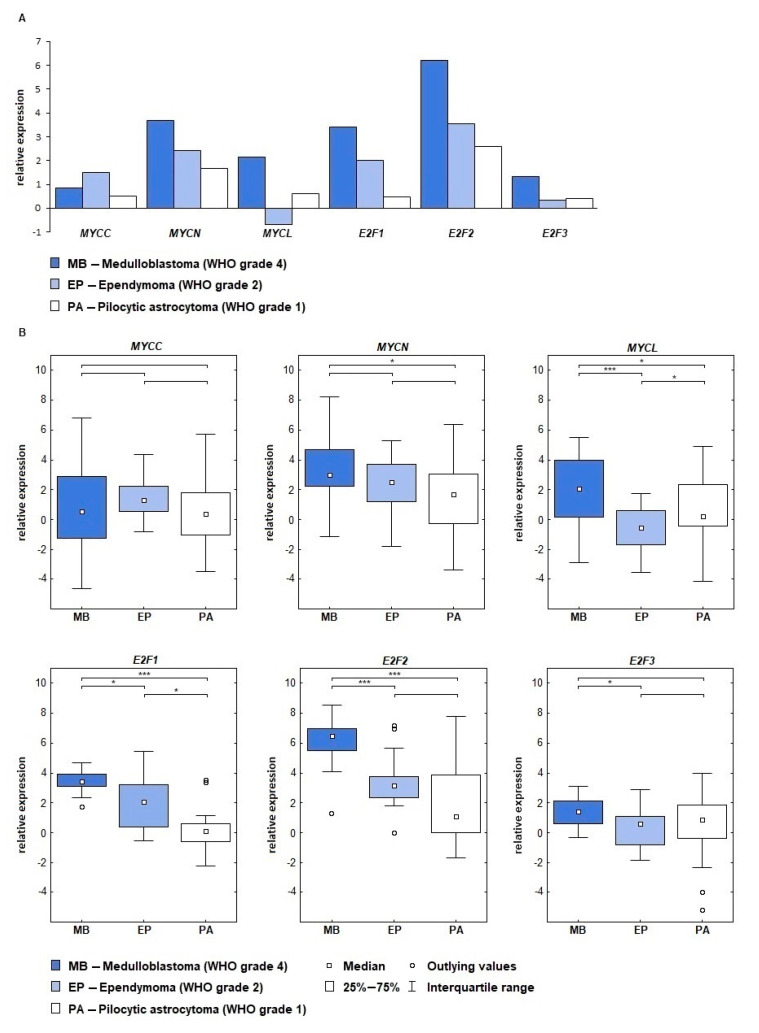
Expression of genes from the *MYC* and *E2F* families in three groups of pediatric brain tumors: medulloblastomas, ependymomas, and pilocytic astrocytomas. (**A**) Relative expression of *MYCC*, *MYCN*, *MYCL*, *E2F1*, *F2F2,* and *E2F3* genes given in ddCt values. (**B**) Relative expression of *MYCC*, *MYCN*, *MYCL*, *E2F1*, *F2F2,* and *E2F3* with an indication of the differences in expression between the groups studied; * *p* < 0.05, *** *p* < 0.001.

Analysis of expression levels of genes from the *MYC* family showed that *MYCN* was characterized by the highest activity (Figure 1). The highest level was confirmed for the medulloblastomas (ddCt = 3.67), which have the highest grade of malignancy among the examined tumors. Differences in expression levels between the analyzed groups showed statistical significance between medulloblastoma (MB) and pilocytic astrocytoma (PA) (*p* = 0.0125, Table 1).

The expression of the *MYCC* gene was at a lower level than *MYCN*. The highest values of *MYCC* were confirmed in the ependymomas (ddCt = 1.49), followed by medulloblastomas, while the lowest expression was showed in the pilocytic astrocytomas, however, no statistically significant differences were found between the groups.

For the *MYCL* gene we obtained the highest expression in the medulloblastomas (ddCt = 2.15), followed by pilocytic astrocytomas. Downregulation was noted in the ependymomas (ddCt = −0.68). The following differences in the expression between the groups *p* = 0.0116,74 (MB vs. PA), *p* = 0.000109 (MB vs. ependymoma (EP)) and *p* = 0.0440,45 (EP vs. PA) were statistically significant.

Gene expression analysis among the members of the *E2F* family showed very high up-expression for the *E2F2* gene. The highest level was found in the medulloblastomas (ddCt = 6.62), followed by EPs and PAs. The differences between the groups for the *E2F2* gene, *p* < 0.000, was noted between MB and PA and MB and EP groups. *E2F1* expression reached a significance level between MBs and PAs (*p* < 0.000), between MBs and EPs (*p* = 0.0135) and between EPs and PAs (*p* = 0.014). The *E2F3* gene obtained the lowest level of expression where ddCt = 1.33 for MBs, ddCt = 0.34 for EPs, ddCt = 0.39 for PAs. Statistically significant differences were noted between MB and EP, where *p* = 0.011.

In conclusion, the *MYCN*, *E2F1*, *E2F2* genes showed the highest expression levels among the studied groups. The highest ddCt values of these genes were noticed in medulloblastomas, followed by ependymomas and pilocytic astrocytomas, indicating a positive correlation between expression level and tumor grade.

### 2.2. miRNA Expression Depends on Tumor’s Histopathology and WHO Grade

**Figure 2:**
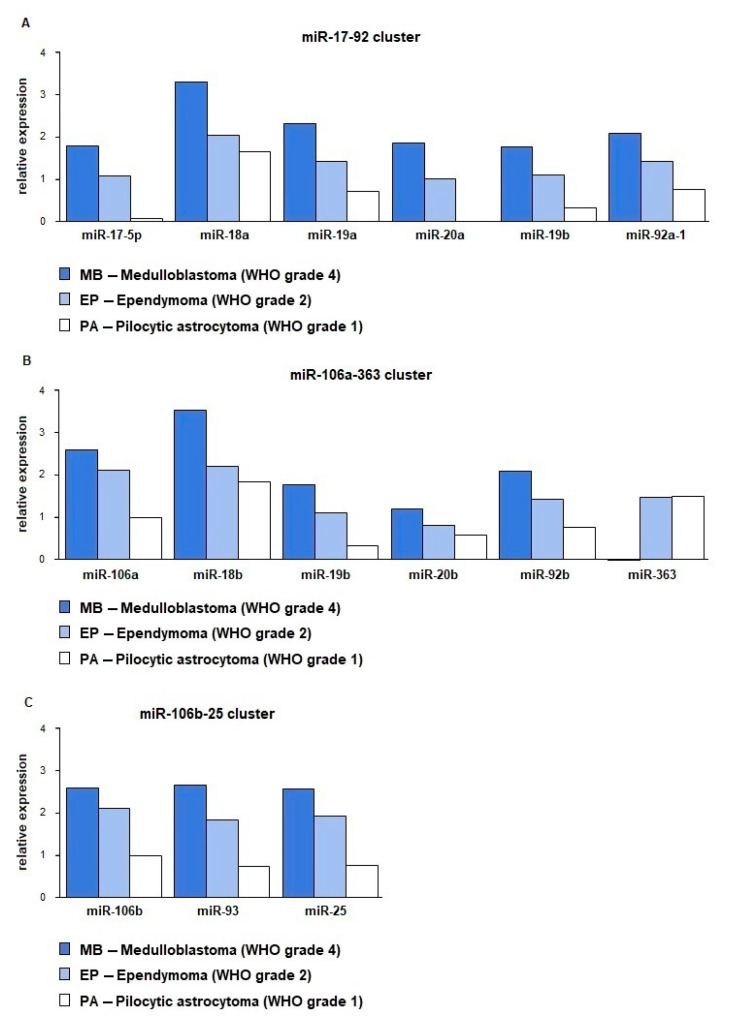
Relative expression level of members of three paralogical clusters in medulloblastoma, ependymoma, and pilocytic astrocytoma. (**A**) miR-17-92 cluster; (**B**) miR-106a-363 cluster; (**C**) miR-106b-25 cluster. Relative expression is given in ddCt value.

**Figure 3:**
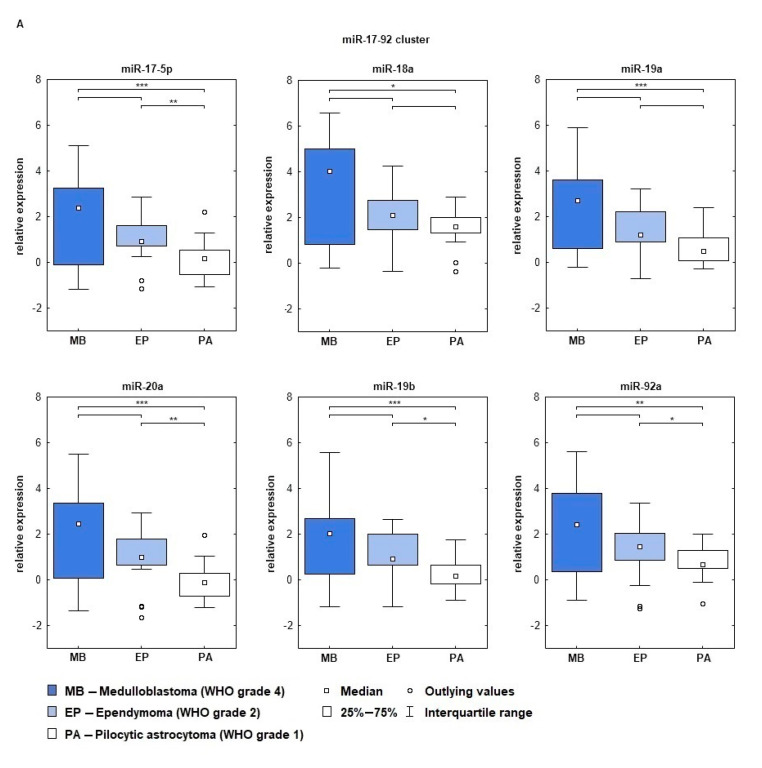

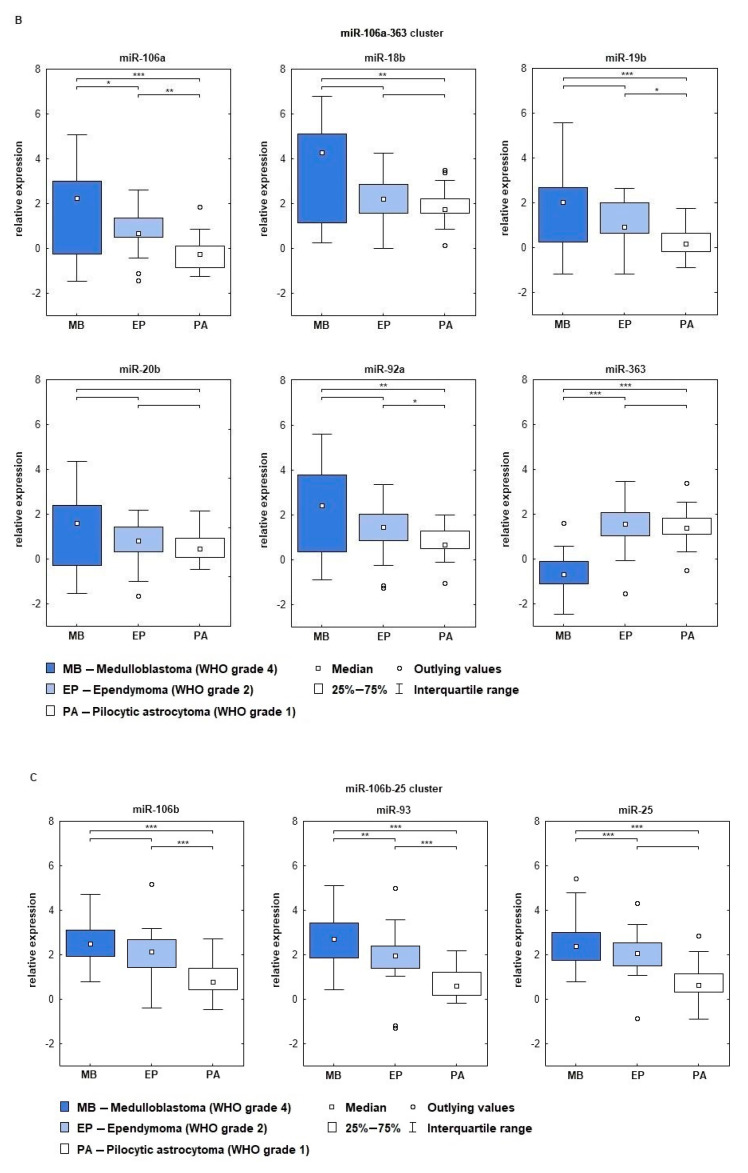
Relative expression of miRNAs from miR-17-92, miR-106a-363, and miR-106b-25 clusters in the analyzed groups of pediatric brain tumors. Relative expression is given in ddCt values. (**A**) miR-17-92 cluster; (**B**) miR-106a-363 cluster; (**C**) miR-106b-25 cluster; * *p* < 0.05, ** *p* < 0.01, *** *p* < 0.001.

Almost all tested miRNAs from miR-17-95, miR-106b-25, and miR-106a-363 clusters showed overexpression in the analyzed cohort of pediatric brain tumors. It has been observed that there is a correlation between miRNA expression and the tumor’s grade. The highest miRNA expression was noted for medulloblastomas, next highest for ependymomas, and finally pilocytic astrocytomas. The highest expression level was confirmed for miR-18a (miR-17-92 cluster) and miR-18b (miR-106a-363 cluster). Double delta Ct of miR-18a was 3.28 for MBs, 2.04 for Eps, and 1.66 for Pas, accordingly. ddCt values noted for miR-18b were 3.54 for MB, 2.19 for Eps, and 1.85 for PAs. It should also be emphasized that miR-17-5p and miR-20-5p achieved very low expression values in pilocytic astrocytomas, ddCt was 0.08 for miR-17-5p, while for miR-20a-5p expression was below the internal control level (−0.11). The one exception was noted for miR-363, for which down-expression in the medulloblastomas (ddCt = −0.70) was showed, while in the ependymomas (ddCt = 1.47) and pilocytic astrocytomas (ddCt = 1.48) the expression was on a higher level. Comparison of miRNA expression levels from the miR-17-92, miR-106b-25, and miR-106a-363 clusters in the three tumor groups showed that the increase of miRNA expression was dependent on WHO grade and type (Figure 2 and Figure 3). Statistically significant differences in miRNA expression occurred between MBs and PAs, next between EPs and PAs, while the smallest differences were noted between MBs and EPs (Table 2).

### 2.3. Relationship between Gene Expression of Genes from MYC and E2F Families and miRNAs

The analysis of correlation between genes from the *MYC* and *E2F* families (Table 3) showed a positive Pearson correlation coefficient in pilocytic astrocytomas, eight out of nine gene–gene pairs achieved statistical significance with r values ranging from 0.55 to 0.81. In the ependymoma group 6 out of nine gene–gene pairs reached the level of statistical significance, r value was in the range of 0.54 to 0.66. In medulloblastomas, only three pairs obtained statistical significance, r value from 0.38 to 0.51. There was no strong correlation between genes and miRNA expression (the correlation coefficient ranged from −0.61 to 0.38). Among the statistically significant results, the most interesting observations concerned the miR-106b-25 cluster. In ependymomas, gene expression negatively correlated with the expression of cluster members, e.g., miR-106b-*MYCC r* = −0.42, miR-106b-*MYCN r* = −0.61, miR-106b-*E2F2 r* = −0.51, miR-106b-*E2F3 r* = −0.58, miR-93-*MYCN r* = −0.47, miR-25-*MYCN r* = −0.47, miR-25-*E2F2 r* = −0.49, miR-25-*E2F3 r* = −0.52. The remaining statistically significant results include miR-363-*E2F3 r* = −0.42 in EPs, miR-92a-*MYCC* r = 0.38 in MBs, miR-92a-*E2F1 r* = −0.40 in MBs, and miR-18b-*E2F1 r* = −0.37 in MBs. No statistically significant results were obtained in pilocytic astrocytomas.

Strong correlations between miRNAs were reported due to the common origin of the miRNAs. The strongest correlation was found in medulloblastomas and ependymomas, while a lower value of the correlation coefficient was noted for pilocytic astrocytomas. Pearson correlation analyses were performed with a 95% confidence interval.

## 3. Discussion

The most important role of miRNA is advanced interactions between particular miRNA groups and genes with crucial cellular functions. One example of such interactions is the feedback loop between *MYC*/*E2F* and miR-17-92. Expression of the *E2F1* gene is caused by MYC and also *MYC* expression is induced by E2F1, forming the positive feedback loop [19]. The expression level of E2F and MYC transcription factors determines further cell activity, including transcription of the members of the miR-17-92 cluster. In addition, MYCC and MYCN can initiate transcription by direct binding to the miR-17-92 promoter [20]. *E2F1* expression is negatively regulated by two miRNAs from the cluster, miR-17-5p and miR-20a [6,21]. Additionally miR-20a modulates *E2F2* and *E2F3* translation [6].

Dysregulated expression of *E2F* and *MYC* families and miR-17-92 cluster are often found in the most types of cancers including lung [9,22], breast [23,24] and prostate tumors [25] or leukemia [26].

Here we present the results of the expression analysis performed for genes from the *MYC* (*MYCC*, *MYCN*, *MYCL*) and *E2F* (*E2F1*, *E2F2*, *E2F3*) families and miRNAs from miR-17-92, miR-106b-25 and miR-106a-363 clusters in three types of pediatric brain tumors showing different histology and grade: medulloblastoma (WHO grade 4), ependymoma (WHO grade 2) and pilocytic astrocytoma (WHO grade 1).

The E2F family consists of E2F1, E2F2, and E2F3 transcription factors, with defined activating function, and E2F4-8, with confirmed inhibiting functions. The first subgroup of E2F proteins are involved in the regulation of the cell cycle and have sufficient transcriptional activity to drive quiescent cells from G1 to S phase [27,28]. MYC family consists of three paralogs MYCC, MYCN, MYCL, which are characterized as known oncogenic factors [29,30].

High levels of E2F1 were described as factors associated with a cell cycle dysregulation. If E2F1 expression is low, the mammalian cell will remain at rest in the G1 phase. In turn, high expression of E2F1 leads to increased cell proliferation that may result in tumor formation and progression [5,31]. Our study confirmed that *E2F1* mRNA levels were correlated with tumor grade and were increased in high grade lesions. Differences between the three analyzed groups revealed a statistically significant level of gene expression with *p* < 0.001 between medulloblastoma and pilocytic astrocytoma and *p* < 0.05 between medulloblastoma and ependymoma, and ependymoma and pilocytic astrocytoma. Upregulation of *E2F1* has been reported in studies performed both on in vitro and in vivo brain tumor models, which described a significant increase in E2F1 expression levels and activity [32,33].

Oliver’s team in research performed on a mouse model of medulloblastoma showed that *MYCN* promotes cell cycle gene expression; increase of *MYCN* expression level was there reported with significantly increased levels of *E2F1* (3.7-fold), and *E2F2* (6.1-fold) [34]. Such observations are consistent with our results. Here we showed the highest expression level of *E2F2*, *MYCN* and *E2F1* in each of the studied groups of tumors (Figure 1A). Swartling and colleagues in their study performed on a medulloblastoma mouse model showed that *MYCN* contributes to tumor initiation and progression. Tumor maintenance requires constant *MYCN* expression, while inhibition of its expression leads to aging of tumor cells [35]. Increase of *MYCN* expression has been reported in cancer with an aggressive course and poor prognosis, particularly that of neural origin, and also in neuroendocrine tumors [30] including medulloblastoma [36], while there is not much evidence linking *MYCN* to glial-derived tumors [37]. In our study, among the three types of tumors examined, the highest expression of *MYCN* was found in medulloblastomas, which confirms the observations made in the previous reports [38]. *MYCN* is a recognized biomarker in neuroblastomas, but little is known about the expression of *MYCN* in less aggressive brain tumors [39]. According to our best knowledge only one report relates to several cases of anaplastic ependymoma (WHO grade 3) [40].

Here we report that *MYCN* showed the highest level of expression in three study groups among the *MYC* family. Our work is based on the analysis of pediatric infratentorial brain tumors, and a high level of *MYCN* expression can be associated with the development of the cerebellum [37]. Additionally MYCC and MYCN proteins are mostly functionally interchangeable [41]. It is very possible that MYCN takes over the MYCC function. In our study, the *MYCC* mRNA level in all groups occurs at a similar level (without statistically significant differences between the groups), while *MYCN* is distinguished by a very high level of expression. High expression of *MYCN* in all groups studied suggests a dependency not on the grade of the tumor but even more on its location. This is consistent with the research presented by Korshunov et al. on pediatric infratentorial glioblastomas with high *MYCN* expression [42].

It was shown that MYCC and MYCN can bind to the promoter of miR-17-92 and initiate transcription [20,43]. Overexpression of miR-92, miR-106a, miR-17-5p, and miR-93 were associated with *MYCN* amplification [44], and in addition, *E2F1* expression is negatively regulated by two miRNAs from the cluster, miR-17-5p and miR-20a [6,21,45]. Therefore we decided to examine the levels of expression of members of the miR-17-92 group and its two paralogs, miR-106a-363 and miR-106b-25. All components of the mir-17-92, mir-106a-363, and miR-106b-25 clusters showed overexpression relative to the control. Two molecules, miR-17-5p and miR-20a, were the most frequently studied and reported miRNAs of these clusters also in brain tumors [46,47]. Studies concerning the expression level of miRNAs in human gliomas have shown that expression of miR-17 and miR-20a were significantly higher than in control tissues. The molecules promoted proliferation and invasion and inhibited apoptosis in glioma cells and thus contributed to increasing malignancy of the tumors [48,49]. Our results showed that miR-17-5p and miR-20a achieved similar levels of expression relative to each other in the individual groups studied; when expression in the medulloblastoma group was high, in ependymoma the level was slightly lower, while in pilocytic astrocytoma these miRNAs reached very low overexpression level compared to the control. This was consistent with the literature reports that the level of expression positively correlates with the malignancy of the tumor [46,47,50]. Moreover miR-20a and miR-17-5p regulated *E2F1* by binding in the 3′-UTR of its mRNA [21]. In our research miR-17-5p and miR-20a did not reach the highest expression among the miRNAs tested, nor did *E2F1* from the *E2F* family of genes. This may indicate the involvement of these factors in mutual regulation. Yang et al. also showed that *E2F1* is a direct target of miR-106a, and the level of miR-106a expression inversely correlates with the tumor grade [51]. In our study we showed that miR-106a expression correlates with the WHO grade, the highest expression level was confirmed in medulloblastoma and the lowest in pilocytic astrocytoma. Such results have also been reported for tumors of glial origin [52,53].

The highest level of expression in our study was noted for miR-18a and miR-18b. miR-18a is highly expressed in many types of cancer and cell lines, enhancing the tumorigenesis, malignancy, and metastatic potential [54,55,56]. In addition, miR-18a plasma concentration was significantly higher in preoperative samples than in postoperative samples in gastrointestinal cancers [55,57]. High expression of miR-18a has also been shown in glioblastoma tissue samples and cell lines. The increasing level of that miRNA was associated with cell proliferation and progression [58,59]. Similar results were obtained for miR-18b. miR-18b is one of the most significantly upregulated miRNAs in colorectal cancer, where miR-18b expression promoted cell proliferation, facilitating cell cycle progression [60]. In breast cancer, overexpression of miR-18b was noted in both clinical samples and cell lines, and upregulated miR-18b increased cell migration [61]. A relationship between miR-18b expression and grade of malignancy was demonstrated; in addition, an increase in miR-18b expression contributed to poor prognosis [60].

Our results demonstrate that miR-18a and miR-18b showed the highest level of expression among all miRNA molecules tested. However, until now there has been no other research on such a large scale covering all elements of the miR-17-92, miR-106b-25, and miR106a-363 clusters, especially in brain tumors in children, therefore it is difficult to answer whether this is a feature unique to this type of lesion. One thing is certain, that miR-18a and miR-18b are found to be high in pediatric brain tumors and that the decrease in expression is associated with lower grade.

miR-363 was the sole miRNA tested by us whose expression level was close to the level of control in medulloblastomas, whereas it was high in ependymomas and pilocytic astrocytomas. The literature concerning this issue is quite limited and concerns mainly glial tumors. Conti et al. in their study conducted on pilocytic astrocytomas (WHO grade 1), diffuse fibrillary astrocytomas (WHO grade 2), anaplastic astrocytomas (WHO grade 3), and glioblastomas (WHO grade 4) showed that miR-363 was upregulated in all the tumors and its level positively correlated with the grade of tested samples [62]. Here we showed that expression of miR-363 was higher in tumors of glial and ependymal rather than embryonal origin, and according to that our results could be confirmation for the observations presented by Conti et al.

The positive Pearson correlation coefficient between the expression of genes from the *MYC* and *E2F* families was observed in the group of pilocytic astrocytomas, then in ependymomas (Table 3), whereas in the medulloblastomas the least pairs gene–gene achieved the results on the level of statistical significance.

Correlation analysis of miRNA-gene pairs expression showed no strong interactions. Only for a few miRNA-gene pairs were the results statistically significant. Among them inverse correlations (*r* > −0.61) were noted in the ependymoma group for miR-106b-*MYCC*, miR-106b-*MYCN*, miR-106b-*E2F2*, miR-106b-*E2F3*, miR-93-*MYCN*, miR-25-*MYCN*, miR-25-*E2F2*, miR-25-*E2F3*, i.e., members of the miR-106b-25 cluster. The *MYC* and *E2F* gene families are involved in the regulation of the cell cycle and their expression levels could be disturbed during carcinogenesis. Moreover, members of the miR-17-92 cluster participate in the regulation of these genes [5,6]. An important feature of miRNA biology is that a single miRNA may be compatible with multiple regions of mRNA, thus regulating entire networks of proteins. Conversely, one mRNA can be targeted by several miRNAs [63]. It should be emphasized that tumorigenesis is a cascade of events, dysregulation of individual genes and miRNA-gene interactions, activation of signaling pathways, which are influenced by multiple factors including tumor type, location, stage, as well as age of the patient [64]. Thus, a gene can be regulated by many miRNAs (*MYC* is predicted to be targeted by 48 miRNAs, according to the database mirdb.org) and it can influence the regulation of other miRNAs by becoming part of a feedback loop. Some of the best characterized feedback loops involving *MYC* are *MYC*/*PTEN*/miR-106b, miR-93, miR-25, miR-19a, miR-22, miR-26a, miR-193b, miR-23b; *MYC*/*RB1*/miR-106a, miR-106b, and miR-17; *MYC*/*VEGF*/miR-106b, miR-106a, miR-93, miR-34a, miR-20a, miR-17, miR-16, miR-15a [65].

To summarize, we confirmed the largest statistically significant differences of miRNA expression between medulloblastomas and pilocytic astrocytomas, followed by ependymomas and pilocytic astrocytomas, while the smallest differences were noted between MBs and EPs. However, we expected the smallest differences between the least malignant tumors of common glial origin, i.e., PAs and EPs. The result may indicate that the levels of miRNA expression depend not only on the grade, but also on tumor type.

Our current research, which focused on the evaluation of miRNA expression from three clusters and related genes from *MYC* and *E2F* families in pediatric brain tumors, demonstrated that expression levels of members of the miR-17-92 cluster and its paralogs are upregulated in the analyzed cohort of cases and levels of their expression correlate with the WHO grade and histology. Members of the *E2F* family were overexpressed in all samples and the highest expression levels were confirmed for *E2F2*. Among the genes from the *MYC* family, the highest expression was observed for *MYCN* and it was also correlated with the WHO grade and type.

Such observation indicates the plausible therapeutic potential of miRNAs as critical targets in brain tumor therapy despite tumor type in the future.

## 4. Materials and Methods

### 4.1. Patients and Tissue Samples

In the analysis 90 samples of childhood brain tumors stabilized in RNA later and stored at −80 °C were included. Brain tumors comprised 30 pilocytic astrocytomas (WHO grade 1), 30 infratentorial ependymomas (WHO grade 2), and 30 medulloblastomas (WHO grade 4). All analyzed tumors were located infratentorially. The age of the patients ranged from 0 to 18 years. Control material constituted Human Brain Total RNA (Invitrogen, cat. No AM7962). The experiments were approved by the Bioethical Committee at the Medical University of Lodz (permit No: RNN/122/17/KE).

### 4.2. RNA Isolation and Reverse Transcription

Total RNA, including a fraction of small non-coding RNAs, was extracted according to the manufacturer’s instructions, using commercially available miRNeasy Mini Kit (Qiagen, Hilden, Germany). The quantity and purity of RNA were analyzed quantitatively and qualitative.

### 4.3. Reverse Transcription and Quantification of Gene Expression by qRT-PCR

cDNA dedicated for gene expression analysis was synthetized from 500 ng of total RNA of each sample by 5x HiFlex Buffer (miScript II RT Kit, Qiagen). The real-time quantitative PCR analysis was performed in duplicate using Fast Advanced Master Mix and specific TaqMan probes (Life Technologies, Carlsbad, CA, USA) for *MYCC* (Hs00153408_m1), *MYCN* (Hs00232074_m1), *MYCL* (Hs00420495_m1), *E2F1* (Hs00153451_m1), *E2F2* (Hs00231667_m1), *E2F3* (Hs00605457_m1) genes and *GAPDH* used as the control housekeeping gene (Hs99999905_m1).

Normalized relative expression levels of the examined gene were calculated in the tested samples compared with control based on the sample’s average Ct value, according the formula in Equation (1):

ddCt = dCt(target sample) − dCt(control sample) = (Ctref_tar_-Ctgene_tar_) − (CTref_cont_-Ctgene_cont_). (1)

### 4.4. Reverse Transcription and Detection of miRNA Expression by qRT-PCR

To conduct miRNA expression analysis 750 ng of total RNA was reverse transcribed using a TaqMan MicroRNA Reverse Transcription kit and specific RT primers from TaqMan MicroRNA assays (Life Technologies, USA) for hsa-miR-17 (assay ID: 0023081), hsa-miR-20a (0005801), hsa-miR-106b (0004421), hsa-miR-93 (0010901), hsa-miR-20b (0010141), hsa-miR-106a (0021691), hsa-miR-18a (0024221), hsa-miR-18b (0022171), hsa-miR-19a (0003951), hsa-miR-19b (0003961), hsa-miR-92a (0004311), hsa-miR-25 (0004031), and hsa-miR-363 (0012711). Two sequences, U6 snRNA (0019731) and hsa-miR-9 (000583) were used as the internal control.

miRNA expression was performed using dedicated TaqMan probes, PCR primer set from TaqMan MicroRNA and Fast Advanced Master Mix (Life Technologies, USA). Reactions for each assay were performed in duplicate, the results were averaged to analyses. CFX96™ Touch Real-Time PCR Detection System was used for acquisition (Bio-Rad, Hercules, CA, USA).

Normalized relative expression levels of the miRNA in the tested samples vs. the control sample were calculated based on the mean Ct value of the sample, according to the formula in Equation (2):

ddCt = dCt(target sample) − dCt(a control sample) = (Ctref_tar_-CtmiRNA_tar_) − (CTref_cont_-CTmiRNA_cont_). (2)

### 4.5. Statistical Analysis

Statistica (v. 13.0) software was used for the statistical analysis of research results. Normality was checked using the Shapiro–Wilk test and the Lilliefors-corrected Kolmogorov–Smirnov. Comparisons of the different miRNA and gene expression levels between groups were performed using ANOVA coupled with Tukey’s post hoc test or the Kruskal–Wallis test, depending on the type of distribution. Spearman’s rank correlation was used to assess the correlations between miRNAs and gene expression.

## Figures and Tables

**Table 1:** *p* values determining the level of statistical significance in gene expression from *MYC* and *E2F* families between the analyzed groups. If *p* value is less than 0.05 we report the result as statistically significant (indicated in bold). MB, medulloblastoma; EP, ependymoma; PA, pilocytic astrocytoma.

Gene	MB vs. PA	MB vs. EP	EP vs. PA
*MYCC*	0.788019	0.471420	0.164877
*MYCN*	**0.012457**	0.375058	0.535525
*MYCL*	**0.011674**	**0.000109**	**0.044045**
*E2F1*	**0.000000**	**0.013521**	**0.014328**
*E2F2*	**0.000000**	**0.000023**	1.000000
*E2F3*	0.132115	**0.010907**	1.000000

**Table 2:** *p* values determining the level of statistical significance in the expression of miRNAs from the miR-17-92, miR-106a-363, and miR-106b-25 clusters between the groups. If *p* value is less than 0.05 we report the result as statistically significant (indicated in bold). MB, medulloblastoma; EP, ependymoma; PA, pilocytic astrocytoma.

	miRNA	MB vs. PA	MB vs. EP	EP vs. PA
17-92	miR-17-5p	**0.0000,66**	1.0000,00	**0.0018,81**
	miR-18a	**0.0112,56**	0.2688,29	0.6894,59
	miR-19a	**0.0002,08**	0.2688,29	0.6894,59
	miR-20a	**0.0000,10**	0.7671,39	**0.0013,14**
	miR-19b	**0.0001,28**	0.0765,68	**0.0343,83**
	miR-92	**0.0030,20**	1.0000,00	**0.0288,40**
106b-25	miR-106b	**0.0001,07**	0.1327,58	**0.0001,70**
	miR-93	**0.0001,07**	0.0076,12	**0.0003,19**
	miR-25	**0.0001,07**	0.0526,61	**0.0002,33**
106a-363	miR-106a	**0.0001,07**	**0.0372,52**	**0.0042,90**
	miR-18b	**0.0083,77**	0.1771,93	0.8113,97
	miR-19b-2 ^A^	**0.0001,28**	**0.0765,68**	**0.0343,83**
	miR-20b	0.0837,45	0.3829,15	0.6841,70
	miR-92-2 ^B^	**0.0030,20**	1.0000,00	**0.0288,40**
	miR-363	**0.0000,00**	**0.0000,00**	1.0000,00

^A^ miR-19b-2 sequential compliance with miR-19b, ^B^ miR-92-2 sequential compliance with miR-92.

**Table 3:** Pearson correlation coefficients between members of the *MYC* and *E2F* families in three analyzed groups. If *p* value is less than 0.05 we report the result as statistically significant (indicated in bold). MB, medulloblastoma; EP, ependymoma; PA, pilocytic astrocytoma.

Gene–Gene	MB	EP	PA
*MYCC-E2F1*	−0.21	**0.55**	**0.75**
*MYCC*-*E2F2*	−0.13	0.30	**0.61**
*MYCC-E2F3*	0.19	**0.54**	**0.72**
*MYCN-E2F1*	**0.40**	**0.66**	**0.81**
*MYCN-E2F2*	**0.51**	**0.60**	**0.79**
*MYCN-E2F3*	−0.21	**0.55**	**0.79**
*MYCL-E2F1*	0.21	0.25	0.37
*MYCL-E2F2*	**0.38**	0.29	**0.55**
*MYCL-E2F3*	−0.22	**0.62**	**0.60**

## Data Availability

The datasets used and analyzed are available from the corresponding author on reasonable request.

## Abbreviations

EP	Ependymoma
MB	Medulloblastoma
PA	Pilocytic astrocytoma
